# Aplastic bone marrow with Cabot rings in blood due to high‐dose olmesartan

**DOI:** 10.1002/jha2.399

**Published:** 2022-02-16

**Authors:** Annemiek M.C.P. Joosen, Luthy S.M. Alcala, Anton A.M. Ermens, Adriaan J. van Gammeren

**Affiliations:** ^1^ Department of Clinical Chemistry and Haematology Máxima Medical Centre Veldhoven The Netherlands; ^2^ Department of Pathology Amphia Hospital Breda The Netherlands; ^3^ Department of Clinical Chemistry and Haematology Amphia Hospital Breda The Netherlands

A 67‐year‐old man with a history of hypertension and chronic obstructive pulmonary disease (COPD) was referred to our hospital because of normocytic anaemia (haemoglobin 100 g/L) and thrombocytopenia (27 × 10^9^/L). There were no fever, weight loss, night sweats, and bleeding tendency. There were no signs of lymphadenopathy or hepatosplenomegaly. The blood smear showed multiple Cabot rings (Figure 1, left). Bone marrow biopsy revealed an aplastic bone marrow without indications for haematological malignancy (Figure 1, right).

**FIGURE 1:**
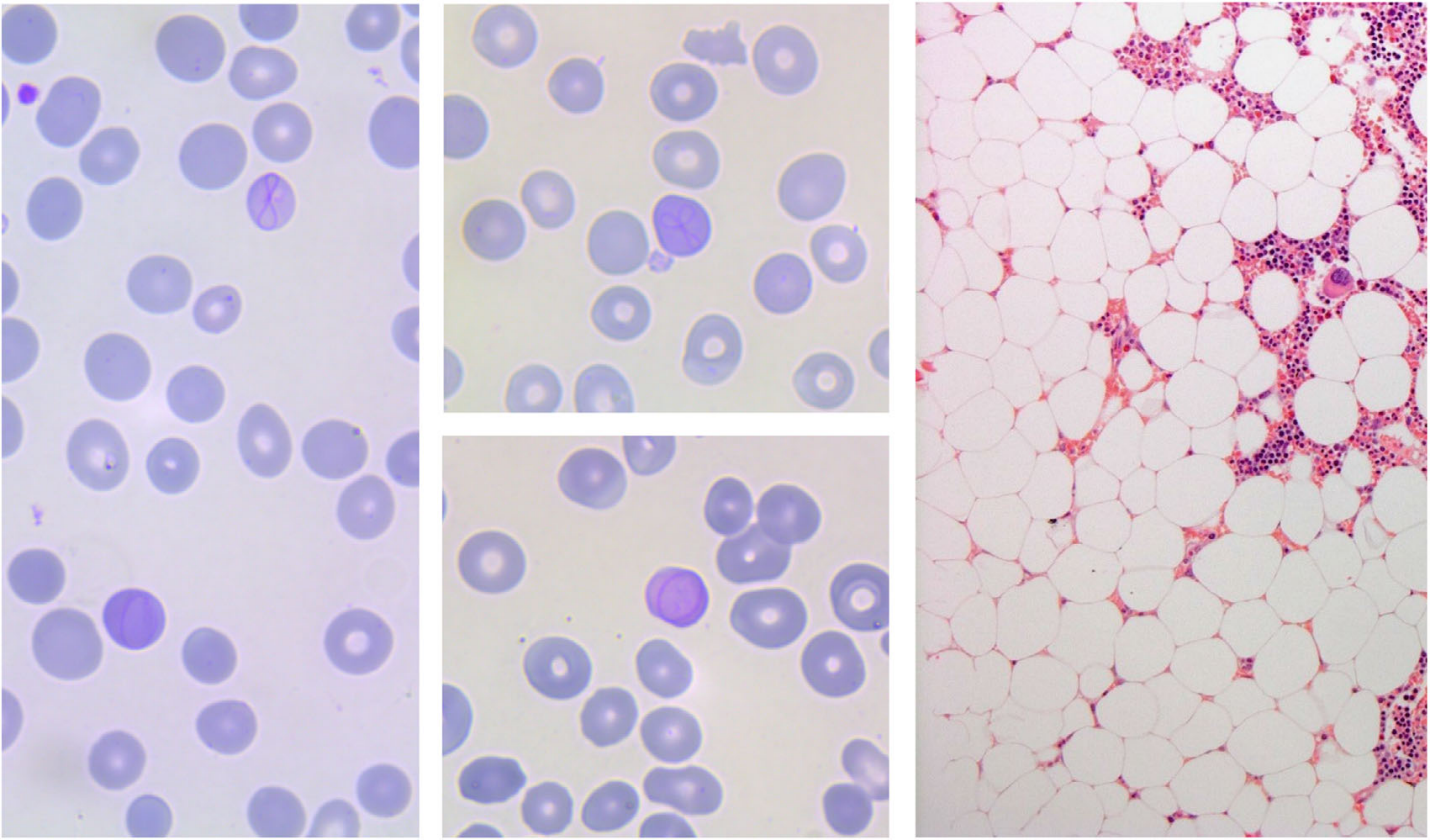
Cabot rings in erythrocytes (left en middle panels) and aplastic bone marrow (right panel) in a patient using a high‐dose olmesartan

The patient admitted to use 160 mg olmesartan a day, while a maximum dose of 40 mg per day is recommended. The patient had been taken this high dose for several months in an attempt to suppress his COPD‐related infections and initially refused to stop. About 9 months later, he was admitted to the intensive care unit with severe pancytopenia and respiratory failure due to an infection. It is unknown whether he used the high dose continuously during this 9‐month period. During his stay in hospital, the olmesartan intake was discontinued and his blood cell counts completely recovered within a few weeks. No more Cabot rings were seen in his peripheral blood.

Cabot rings are likely remnants of the mitotic spindle. Cabot rings is a rare finding in dyserythropoiesis, megaloblastic anaemia, myelodysplasia, and can occur with some medications. As there was no evidence for underlying disease or vitamin deficiency, the anaemia and thrombocytopenia and Cabot rings were attributed to the high intake of olmesartan.

## AUTHOR CONTRIBUTIONS

A.A.M. Ermens examined the peripheral blood, L.S.M. Alcala examined the bone marrow, A.A.M. Ermens and L.S.M. Alcala provided the photos, A.M.C.P. Joosen and A.J. van Gammeren searched the literature and wrote the paper.

## CONFLICT OF INTEREST

The authors declare no conflict of interest.

